# Preventive Therapeutics

**Published:** 1890-02

**Authors:** 


THE field of preventive therapeutics is steadily growing, as can be seen from the many gratifying pharmacal innovations in the line of antiseptic agents. The latest acquisition in this respect is one which is intended to benefit more the attending physician and relatives of the patient, than the patient himself, viz., antiseptic cologne. The preparation is gotten up in bottles on the principle of the cologne atomizers, and is really a useful and convenient agent. The physician is supposed to carry a miniature bottle of antiseptic *eau-de-cologne* about his person and to sprinkle his clothes and person with this agreeable and at the same time germicidal solution before leaving the sick room. The relatives of the patient, on the other hand, can use the cologne for the floor, furniture, bed-clothes, urine of the patient, etc.—Correspondence, *Medical and Surgical Reporter.*

